# Catastrophic Antiphospholipid Syndrome: A Rare but Life‐Threatening Thrombotic Storm—A Case Report and Literature Review

**DOI:** 10.1155/crrh/8852169

**Published:** 2026-03-02

**Authors:** Renee Morecroft, Jordan Phillipps, Vikas Majithia, Sehreen Mumtaz

**Affiliations:** ^1^ Department of Internal Medicine, HCA Florida Orange Park Hospital, Orange Park, Florida, USA; ^2^ Department of Dermatology, Mayo Clinic, Jacksonville, Florida, USA, mayo.edu; ^3^ Department of Rheumatology, Mayo Clinic, Jacksonville, Florida, USA, mayo.edu

**Keywords:** antiphospholipid antibodies, case report, catastrophic antiphospholipid syndrome, thrombotic microangiopathy

## Abstract

Catastrophic antiphospholipid syndrome (CAPS) is a rare, life‐threatening variant of antiphospholipid syndrome (APS) characterized by rapid, widespread thrombosis leading to multiorgan failure. Affecting less than 1% of APS patients, CAPS is associated with a high mortality rate of 30%–50%, necessitating prompt diagnosis and aggressive treatment. The mainstay of management includes anticoagulation, high‐dose glucocorticoids, and plasma exchange or intravenous immunoglobulins, with biologic therapies such as rituximab and eculizumab reserved for refractory cases. We report a case of a 60‐year‐old male with a history of triple‐antibody–positive APS complicated by recurrent diffuse alveolar hemorrhage (DAH), adrenal hemorrhage, chronic kidney disease, and superficial vein thrombosis. His condition progressed to CAPS approximately 5 years prior with a course complicated by heparin‐induced thrombocytopenia. His condition stabilized with high‐dose corticosteroids and rituximab therapy with sustained symptomatic improvement after 10 months of rituximab. This case highlights the complexity of CAPS diagnosis and management, in the context of DAH, emphasizing the importance of early recognition, multidisciplinary care, and individualized treatment strategies. Our patient’s prolonged disease stabilization with rituximab underscores its potential role in long‐term CAPS management. Further research is needed to refine treatment protocols and improve outcomes for this rare but life‐threatening condition.

## 1. Introduction

Catastrophic antiphospholipid syndrome (CAPS) is a rare, life‐threatening variant of antiphospholipid syndrome (APS) characterized by rapid onset of widespread thrombosis, leading to multiorgan failure. It occurs in < 1% of APS patients and predominantly affects females, with a mean age of onset around 38 years. CAPS typically involves multiple organ systems, including renal (71%–73%), pulmonary (60%–66%), cerebral (56%–60%), cardiac (50%–52%), and cutaneous (47%) manifestations [1]. Diagnosis is based on clinical criteria, including evidence of rapid‐onset multiple organ involvement, histopathological confirmation of small‐vessel occlusions, and laboratory detection of antiphospholipid (aPL) antibodies [2]. Common triggering factors include infections, surgery, and malignancy [2]. Despite advances in treatments, CAPS carries a poor prognosis, with a mortality rate of approximately 30%–50%. Early recognition and aggressive intervention are crucial for improving outcomes. The cornerstone of treatment is “triple therapy,” consisting of anticoagulation, high‐dose glucocorticoids, and plasma exchange (and/or intravenous immunoglobulins). In refractory cases, rituximab and eculizumab may be considered [3]. Here, we present a unique case of CAPS that contributes to the CAPS registry, a comprehensive database providing valuable insights into epidemiology, clinical presentation, diagnosis, prognosis, and treatment options for this rare but life‐threatening condition.

## 2. Case Summary

**FIGURE 1:**
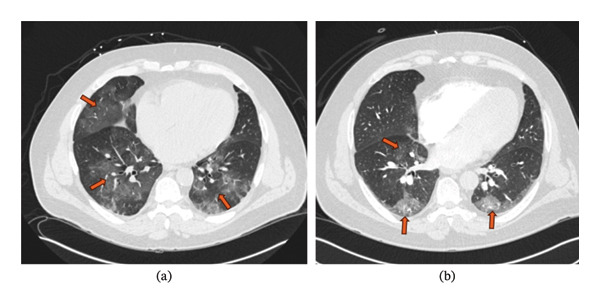
CT chest displaying several recurrences of diffuse alveolar hemorrhage in the present case. (a) 3 years ago—new, bilateral, multifocal, patchy ground‐glass densities, predominantly in broncho‐centric distribution, are scattered in both lungs. (b) 2 years ago—new, multifocal, patchy bronchovascular ground‐glass opacities throughout the lungs with more confluent ground‐glass in the posterior lower lobes. In the context of chest pain, hemoptysis, and known APLS, these findings are consistent with diffuse alveolar hemorrhage.

A 60‐year‐old male with a past medical history of gout and chronic kidney disease presented to our rheumatology clinic for further evaluation of known APS. Approximately 20 years before his current presentation, he experienced a superficial vein thrombosis, prompting a coagulopathy evaluation that revealed positive aPL antibodies (anti‐β2 glycoprotein I 73 GPI IgG units; anticardiolipin 22 GPL U/mL), leading to the initiation of warfarin therapy. Five years ago, he was hospitalized for atrial fibrillation requiring cardioversion, after which he developed heart failure, DAH, stroke, and bilateral adrenal hemorrhage—findings consistent with CAPS. At that time, he tested triple positive for aPL antibodies (elevated β2 glycoprotein IgG [49.6 SMU], negative β2 glycoprotein IgM [< 9.4 SMU], negative anti‐phospholipid IgM [< 9.4 SMU], positive antiphospholipid IgG [20.2 GPL], and positive lupus anticoagulant) and was also diagnosed with heparin‐induced thrombocytopenia. Two years later, he experienced recurrent DAH, requiring treatment with high‐dose corticosteroids and rituximab. Despite multiple attempts to taper his regimen, he had two additional DAH recurrences in subsequent years with CT chest imaging on those occasions showing bilateral, multifocal, patchy ground‐glass densities predominantly in broncho‐centric distribution in both lungs (Figure 1). DAH was also confirmed by bronchoalveolar lavage showing hazy bloody return and an elevated white blood cell count, with absolute neutrophil counts exceeding 1000/μL on both occasions. This necessitated escalation of therapy with high‐dose corticosteroids and an increased rituximab dose (1000 mg ×2 every six months). At his most recent follow‐up (approximately 4 years after his initial CAPS presentation and 1.5 years after his last DAH episode), he reported persistent symptomatic improvement, with no hemoptysis or signs of disease progression. Given his reduced immunoglobulin levels and the absence of APS‐related events while on rituximab (2 infusions of 1000 mg every 6 months) for the past 10 months, plans were made to taper his rituximab regimen to 1000 mg ×1 every 6 months. During this time, he continued routine follow‐ups with ophthalmology (annual retinal exams), endocrinology (for adrenal insufficiency), and strict therapeutic anticoagulation monitoring as appropriate.

## 3. Discussion

CAPS was first described by Asherson in 1992 and involves widespread microvascular thrombosis and multiorgan failure [4]. Unlike classical APS, which causes isolated thromboses, CAPS presents as a “thrombotic storm” requiring urgent treatment. Our study contributes a unique case to the CAPS registry and includes a concise literature review (Table 1) addressing CAPS epidemiology, pathophysiology, clinical features, diagnosis, and management.

**TABLE 1:** Summary of the existing CAPS literature.

Study	Objective	Methods	Results	Conclusion
Rodríguez‐Pintó et al., [1]	Analyze clinical and immunologic manifestations of CAPS patients from the CAPS registry	Descriptive analysis of 500 patients from the CAPS registry	69% female, mean age 38 ± 17 years, 40% with associated autoimmune disease (mainly SLE), 65% triggered by infections, multiorgan involvement, 37% mortality	CAPS presentation varies by age and underlying disease; infections are a common trigger
Cervera et al., [2]	Describe clinical and laboratory features, precipitating factors, treatment, and outcome of CAPS patients	Analysis of 280 patients from the CAPS registry	72% female, mean age 37 ± 14 years, 46% primary APS, 40% SLE, 53% with precipitating factors, 44% mortality, best outcomes with anticoagulants + corticosteroids + plasmapheresis/IVIG	CAPS is life‐threatening but can be managed with combination therapy
Asherson et al., [5]	Analyze clinical and laboratory characteristics of CAPS patients	Review of 50 patients (5 from clinics, 45 from literature)	66% female, mean age 38 ± 14 years, 56% primary APS, 30% SLE, 22% with precipitating factors, 50% mortality, best outcomes with anticoagulation + steroids + plasmapheresis/IVIG	CAPS involves multiorgan failure and high mortality, but combination therapy improves outcomes
Çelikdelen and Bilici [6]	Highlight the high mortality risk of CAPS	Case report	Fatal outcome of a 33‐year‐old male with CAPS and rapid clinical decline despite anticoagulation, corticosteroid, immunosuppressive, and IVIG therapy	CAPS has a high mortality risk
Ruffatti et al. [7]	Evaluate the clinical significance of laboratory findings and report treatment effects in CAPS patients	Analysis of 14 CAPS cases treated at a single center	Triple aPL antibody positivity prevalent, higher IgG anticardiolipin and anti‐β2Glycoprotein I titers, all patients recovered with triple therapy (anticoagulation + PE + steroids)	Specific aPL antibody profile is a risk factor; early triple therapy is effective
Berman et al. [8]	Describe the clinical manifestations, laboratory features, and outcomes of rituximab‐treated CAPS	Systematic review of 20 patients in the CAPS registry	15 cases successfully recovered with a 75% recovery rate	Rituximab is a viable option for treating refractory CAPS
Lopez‐Benjume et al. [9]	Describe the real‐world experience of eculizumab use in CAPS	Systematic review of 39 patients in the CAPS registry	29 cases successfully recovered with a recovery rate of 74.4%	Eculizumab is a viable option for treating refractory CAPS, especially in the setting of complement‐mediated thrombotic microangiopathy
Berman et al. [10]	Describe clinical characteristics, laboratory features, treatment, and outcome of pediatric CAPS patients	Analysis of 45 pediatric patients from the CAPS registry	71.1% female, mean age 11.5 ± 4.6 years, 68.9% primary APS, 28.9% SLE, 60.9% with infections as a precipitating factor, 26.1% mortality	Pediatric CAPS has higher infection‐related triggers and lower mortality compared to adults

*Note:* CAPS = catastrophic antiphospholipid syndrome, IVIG = intravenous immunoglobulin, and aPL = antiphospholipid.

Abbreviation: SLE, systemic lupus erythematosus.

CAPS is rare (1 in 5 million/year) but severe and may be the initial APS presentation. It affects more women, often with primary or SLE‐associated APS [11]. Males, though fewer, have worse outcomes and higher mortality, often due to cardiac complications [1, 5]. Additionally, the study by Asherson et al. (1998) also noted that males with CAPS had a significant mortality rate, with cardiac complications being a major cause of death. Triggers include infections (65%), surgery/trauma (15%), pregnancy (22%), subtherapeutic INR (68%), and certain medications [1, 12].

CAPS is driven by aPL antibodies—especially anti‐β2GPI—which induce endothelial damage, platelet activation, and thrombin generation [11]. Complement activation is a key mechanism, leading to cytokine storms and thrombotic microangiopathy (TMA), distinct from classic APS [11]. CAPS follows a two‐hit model: chronic aPL presence and a trigger. Genetic defects in complement regulation also predispose to CAPS [11, 13]. Unlike classic APS, which primarily involves isolated medium‐to‐large vessel thrombosis, CAPS is marked by widespread microvascular thrombosis, thus resembling thrombotic thrombocytopenic purpura (TTP) and hemolytic uremic syndrome (HUS).

Diagnosis follows the 2023 ACR/EULAR criteria, with “definite” CAPS requiring four features: (1) ≥ 3 organ involvement, (2) simultaneous onset, (3) small‐vessel occlusion on biopsy, and (4) aPL positivity for ≥ 12 weeks [11]. “Probable” CAPS is diagnosed if all four criteria are met, but only two organs/systems and/or sites are involved [11]. Our patient presented simultaneously with DAH, stroke, and bilateral adrenal hemorrhage. He likely met definite CAPS criteria given the microthrombosis associated with DAH and adrenal hemorrhage though not histologically proven. Additionally aPL antibodies were also present on two separate occasions. Renal injury (71%–73%) shows TMA and neurologic symptoms (56%–60%), including encephalopathy and stroke [11, 13]. Cardiac (50%–52%), cutaneous (47%), and hematologic involvement, including DIC and HELLP syndrome in pregnancy, are also common [11, 13]. Notably, while thrombocytopenia is uncommon among high‐risk APS patients, a decline in platelet count has been observed universally in those who progress to CAPS, suggesting that new or worsening thrombocytopenia in this population should be regarded as an early warning sign of catastrophic transformation [14]. Cutaneous manifestations occur in 47% of cases and include livedo reticularis, livedo racemosa, purpura, skin necrosis, and Raynaud’s phenomenon [11, 13].

Pulmonary complications occur in 60%–66% of CAPS cases and include pulmonary embolism (26%), diffuse alveolar hemorrhage (DAH) (12%), acute respiratory distress syndrome (ARDS) (36%), and pulmonary edema (8%) [11, 13, 15]. A descriptive analysis of the CAPS registry reported pulmonary embolism in 48.6% of episodes, DAH in 28.3%, and both in 2.6% [15]. DAH pathogenesis in CAPS, as observed in this case, is multifactorial—driven by microvascular thrombosis, TMA, and, at times, direct involvement of pulmonary capillaries. Bronchoalveolar lavage and lung pathology, though rarely performed, revealed heterogeneous pulmonary involvement, encompassing both thrombotic (PE and TMA) and nonthrombotic inflammatory patterns (DAH) [15]. Notably, DAH was significantly associated with laboratory evidence of TMA, triple‐positive aPL antibodies, and hypocomplementemia. Based on these findings, three pulmonary patterns in CAPS were proposed: (1) PE, (2) DAH with systemic TMA and hypocomplementemia, and (3) DAH without systemic TMA, with or without hypocomplementemia. These distinctions underscore the importance of thorough diagnostic evaluation to differentiate between thrombotic and hemorrhagic complications, as treatment approaches differ.

Several other CAPS cases are reported in the literature (Table 1). One case involved a young male who died from CAPS complicated by DAH, acute renal failure, and recurrent thromboses despite anticoagulation, corticosteroids, IVIG, and immunosuppression [6]. Clinical analyses of the CAPS registry identify infections and autoimmune diseases as the most common triggers [1, 2]. Reviews by Cervera et al., Asherson et al., and Ruffatti et al. show that triple therapy yields the best outcomes, though mortality remains high (44%–50%) [2, 5, 7]. Registry data also show a 75% recovery rate in 15 of 20 rituximab‐treated cases and 74.4% in 29 of 39 eculizumab‐treated patients [8, 9]. Our case adds to the growing evidence supporting rituximab in refractory CAPS, with both rituximab and eculizumab offering promising options for difficult‐to‐treat cases.

CAPS management primarily involves triple therapy: anticoagulation, high‐dose steroids, and plasma exchange or IVIG. Heparin is the first‐line anticoagulant, with warfarin (target INR 3–4) used for long‐term management [3, 16]. Adjunctive agents such as statins and hydroxychloroquine provide additional anti‐inflammatory and antithrombotic benefits [16]. High‐dose methylprednisolone, plasma exchange, and IVIG help modulate the immune response [3]. Refractory cases may require targeted therapies such as rituximab (B‐cell depletion) or eculizumab (complement inhibition) [3]. Treating hemorrhagic pulmonary complications, such as DAH, poses particular challenges due to the need to balance thrombosis prevention with the risk of life‐threatening bleeding [17]. Management must be individualized and may involve temporary reduction or cessation of anticoagulation, alongside intensified immunosuppression. Decisions regarding anticoagulation should be made in a multidisciplinary setting, carefully weighing the risks of recurrent thrombosis versus uncontrolled hemorrhage. Given the limited high‐quality evidence, expert consultation is strongly recommended.

CAPS is a rare but life‐threatening thrombotic disorder requiring prompt recognition and aggressive treatment. This case underscores its rapid, multisystem progression, and diagnostic complexity, highlighting the need for high clinical suspicion in APS patients with sudden widespread thrombosis. It also reinforces the growing role of targeted therapies—such as complement inhibitors and B‐cell depletion—in managing severe cases. Our report contributes a unique case to the CAPS registry and includes a focused literature review for context. Continued research is essential to refine biomarker‐based risk assessment and personalize treatment to improve survival and long‐term outcomes.

**Nomenclature**
CAPS: Catastrophic antiphospholipid syndromes
APS: Antiphospholipid syndrome
DAH: Diffuse alveolar hemorrhage
SLE: Systemic lupus erythematosus
INR: International nationalized ratio
aPL: Antiphospholipid
β2GPI: β2‐Glycoprotein I
NET: Neutrophil extracellular traps
TTP: Thrombotic thrombocytopenic purpura
HUS: Hemolytic uremic syndrome
DIC: Disseminated intravascular coagulation
HELLP: Hemolysis, elevated liver enzymes, and low platelets
IVIG: Intravenous immunoglobulin
LMWH: Low–molecular‐weight heparin

## Funding

This article has no funding source.

## Consent

A written informed consent was obtained from the patient for publication of this case report and any accompanying images.

## Conflicts of Interest

The authors declare no conflicts of interest.

## Data Availability

Data sharing is not applicable to this article as no datasets were generated or analyzed during the current study.
